# Virus Shedding of Avian Influenza in Poultry: A Systematic Review and Meta-Analysis

**DOI:** 10.3390/v11090812

**Published:** 2019-09-02

**Authors:** Evelien A. Germeraad, Pim Sanders, Thomas J. Hagenaars, Mart C.M. de Jong, Nancy Beerens, Jose L. Gonzales

**Affiliations:** 1 Department of Virology, Wageningen Bioveterinary Research (WBVR), P.O. Box 65, 8200 AB Lelystad, The Netherlands; 2 Department of Bacteriology and Epidemiology, WBVR, P.O. Box 65, 8200 AB Lelystad, The Netherlands; 3 Quantitative Veterinary Epidemiology, Wageningen UR, P.O. Box 338, 6700AH Wageningen, The Netherlands

**Keywords:** avian influenza, virus shedding, poultry, systematic review, meta-analysis

## Abstract

Understanding virus shedding patterns of avian influenza virus (AIV) in poultry is important for understanding host-pathogen interactions and developing effective control strategies. Many AIV strains were studied in challenge experiments in poultry, but no study has combined data from those studies to identify general AIV shedding patterns. These systematic review and meta-analysis were performed to summarize qualitative and quantitative information on virus shedding levels and duration for different AIV strains in experimentally infected poultry species. Methods were designed based on the Preferred Reporting Items for Systematic Reviews and Meta-Analyses (PRISMA) guidelines. Four electronic databases were used to collect literature. A total of 1155 abstract were screened, with 117 studies selected for the qualitative analysis and 71 studies for the meta-analysis. A large heterogeneity in experimental methods was observed and the quantitative analysis showed that experimental variables such as species, virus origin, age, inoculation route and dose, affect virus shedding (mean, peak and duration) for highly pathogenic AIV (HPAIV), low pathogenic AIV (LPAIV) or both. In conclusion, this study highlights the need to standardize experimental procedures, it provides a comprehensive summary of the shedding patterns of AIV strains by infected poultry and identifies the variables that influence the level and duration of AIV shedding.

## 1. Introduction

Avian influenza viruses (AIV) belong to the family Orthomyxoviridae, genus *influenza virus A*. There are several subtypes of AIV, which can be distinguished by two surface glycoproteins: Hemagglutinin (HA) and neuraminidase (NA). Currently sixteen HA and nine NA subtypes have been identified in birds [1]. AIV are also classified based on their pathogenicity in chickens. Less virulent types of AIV, low pathogenic avian influenza virus (LPAIV), cause asymptomatic or (sub)clinical infections with mild to severe respiratory disease [2]. During infections with a highly pathogenic avian influenza virus (HPAIV), the more virulent type of AIV, chickens shows severe systematic clinical signs and high mortality rates are present in the flock. The natural reservoir of AIV is the waterfowl, which is able to transmit the virus to poultry via direct contact [1,2]. From the start of the 21st century, HPAIV has been an important threat for poultry all over the world and has led to great economic losses in the poultry sector due to morbidity, mortality and culling of infected poultry [3,4]. In addition to the effects in poultry, some AIV strains are able to infect humans, e.g., 860 human cases, of which 454 are deaths, caused by H5N1 AIV were reported to the WHO from 2003–2018 [5]. Besides, AIV are suspected to become the precursor viruses of a pandemic in humans [6,7].

Since AIV, especially HPAIV, has a major impact on poultry, much research is focusing on obtaining more knowledge on the infectiousness and transmission potential of this pathogen. Indirect indicators of the infectiousness of an infected bird are the level and duration of virus shedding. These parameters are often used to obtain information of the infection characteristics and the transmission potential of the virus to susceptible recipient hosts during challenge and transmission experiments. The level and duration of virus shedding was studied in different experimental settings, designed to answer a variety of research questions. First of all, shedding can be used to determine which poultry species, age groups or breeds could be potentially more infectious following infection [8,9,10,11]. The occurrence of shedding can also be an indication of virus transmission and effective inoculation [12] and thus can be used for dose-response studies of AIV [13]. Challenge experiments can be used to assess the risk of infection and transmission of new AIV isolates from wild birds to poultry. When poultry start to shed the virus after inoculation with a virus derived from wild birds, it may indicate that the specific virus is able to first infect poultry and possibly transmit between poultry [14]. To actually quantify transmission and the effect of vaccination, experiments are absolutely necessary [15,16]. Finally, shedding is used as a parameter for the evaluation of intervention measures against AIV, such as vaccination, where a reduction in shedding levels and duration could be considered as an indicator of vaccine efficacy in the reduction of infectiousness [17,18,19,20].

In general, a meta-analysis of small-scale animal experiments has the potential of enhanced statistical power, obtained by combining the observations of a set of studies. However, in the case of virus shedding, comparison of the published information is complicated by the fact that different experimental approaches were used. The methodology differs mainly in: (1) The use of different hosts: Species, ages and breeds, (2) the use of different virus strains and (3) the experimental design: Inoculation doses and routes, the types of samples and interval of sampling during the experiment.

The objective of this systematic review (SR) is to get a better understanding of the characteristics of AIV shedding in poultry. Therefore, information on experimental methods was collected and summarized to obtain qualitative information of experimental approaches to study virus shedding in poultry. Subsequently, a quantitative meta-analysis was performed, in which variables that influence both virus shedding levels and duration of different AIV strains were quantitatively identified, whilst correcting for experimental biases.

## 2. Methods

### 2.1. Systematic Review Methodology

A SR uses pre-specified and standardized methods to identify and critically appraise the relevant research, and to collect, report and analyze data from the studies that are included [21,22]. This SR followed the Preferred Reporting Items for Systematic Reviews and Meta-Analyses (PRISMA) guidelines [23], with modifications where needed as the PRISMA statement refers mainly to human intervention studies. The PRISMA review methodology provides key guidance on limiting publication bias, including quality assessment and transparency. The SR consisted of four steps: (1) Literature search, (2) screening and quality assessment, (3) data extraction and (4) data analysis and summation. Before the start of the literature search, a protocol was developed to define the different variables and outcomes for this SR. The protocol is provided as Table S1.

### 2.2. Literature Search

The purpose of the literature search was to identify data on virus shedding levels and the duration of AIV in poultry at a high sensitivity, capturing all relevant citations. Literature was searched in the electronic database PubMed and the Ovid search engine. Using the Ovid search engine CAB Abstracts, Biological Abstracts and AGRICOLA databases were searched simultaneously. No restrictions were imposed on publication date. The last search was performed on 19th July 2017.

To find all relevant search terms related to AIV shedding in the databases, the Mesh database of PubMed was visited. Furthermore, G. Koch and J. L. Gonzales, both AIV experts in respectively virology and veterinary epidemiology, working at Wageningen Bioveterinary Research (WBVR), were consulted. Boolean operators AND and OR were used to narrow down the search. Table S2 presents an overview of the search terms used for the database search. Next to database searches, references of papers that passed the first screening were screened for relevant citations and added to the database in Endnote if not yet present. Applicable grey data were added to enlarge the dataset. The electronic PubMed data collection tool automatically removed duplicate citations of the PubMed search. Duplicate citations of the Ovid search were removed in Endnote with the available tool and checked by hand. The literature search was executed by one reviewer.

### 2.3. Screening and Quality Assessment

After searching the literature, the titles and abstracts of the publications were screened. The first literature screening consisted of basic criteria, while the second screening assessed the quality of the studies that passed the first screening. The screening was performed by one reviewer, in case of doubt a second reviewer was consulted. As a result of the low specificity of the literature search, the aim of the first screening was to quickly remove non-relevant citations. Primary research studies written in English were assessed, no restrictions were imposed on the publication date. The study population evaluated consisted of healthy poultry of all ages, experimentally infected with AIV. Only experimentally infected poultry was considered, as infection conditions are unknown for poultry and wild birds naturally infected. Studies experimentally evaluating virus shedding of AIV were assessed. In addition, studies evaluating the effect of either experimental or commercial vaccination on virus shedding were considered, as the challenge control group could provide data for the meta-analysis. Types of outcomes include virus shedding level (EID_50_/mL, TCID_50_/mL, viral RNA copies and PCR cycle thresholds from real time PCR) and virus shedding duration (days). The first screening aimed to identify relevant studies, the following criteria were used:

The publication is written in English;
The study described is primary research;
Influenza A virus is the subject being studied;
The study was performed in poultry;
Virus shedding is quantified.

Studies that did not meet all the mentioned criteria did not advance to the second screening. If it was unclear from the abstract whether all criteria were met, the study did proceed to the next screening to ensure high sensitivity. The purpose of the second level screening was to identify studies of sufficient quality that quantify AIV shedding. Full texts of studies remaining after first screening were screened for the following criteria:

All criteria of the first screening are met;
The subtype of the virus being studied is known;
The pathogenicity classification of the virus (LPAIV or HPAIV) is known;
The experimental units are confirmed negative for AIV in advance of the study;
The virus inoculation route and dose are described;
Virus shedding is quantified by PCR or by virus isolation;
The sampling interval of virus shedding is described or;
Peak virus shedding is described or can be determined from the data.

A study was included if all criteria were described. Studies that did not meet all criteria were considered as qualitatively insufficient or irrelevant. Screening was executed by one reviewer, however, in the case of doubt a second reviewer was consulted.

### 2.4. Data Extraction

If all criteria of the screening were met and the article passed quality assessment, data was extracted and added to a database. This database was developed by two reviewers and one reviewer executed the data extraction.

Qualitative and quantitative data were both extracted. Qualitative data, which was extracted for each experiment, included: (1) AIV subtype (including the clade if applicable); (2) infected poultry species (including further species information e.g., layer/broiler); (3) pathotype (HPAIV or LPAIV); (4) age at infection (in days); (5) inoculation route and dose; (6) type of samples and (7) host origin of the AIV (the species from which the AIV originates). Daily shedding levels were extracted as quantitative data, which were used to determine peak shedding, average shedding and shedding duration. The geometric mean was used to represent average virus shedding, because a geometric mean is less sensitive to outliers than an ordinary arithmetic mean. If data was only reported in figures, the PlotReader tool [24] was used to extract data. Daily shedding had to be quantified in one of the following units: EID_50_, EID_50_-equivalents, TCID_50_, TCID_50_-equivalents, plaque forming units (PFU), viral RNA copies or cycle thresholds (ct). Studies reporting EID_50_ or TCID_50_ equivalents used a standard curve, based on calibration controls containing known amounts of virus, for conversion of ct to EID_50_-equivalents or TCID_50_-equivalents.

### 2.5. Data Analysis and Summation

The analysis consisted of two parts: A qualitative and a quantitative analysis (meta-analysis).

#### 2.5.1. Qualitative Analysis

The qualitative analysis identified and described different biological and experimental characteristics assumed to influence AIV shedding e.g., poultry species, age of poultry, inoculation routes, type of samples that were collected, outcome units and virus subtypes used in the included studies. In addition, the qualitative analysis shows the heterogeneity in the experimental approaches observed in the selected studies.

#### 2.5.2. Quantitative Analysis (Meta-Analysis)

The quantitative analysis described the relationship between several explanatory variables and virus shedding levels and duration of AIV. Data used to characterize virus shedding patterns were: (1) geometric mean virus shedding per unit sample (mL−1; geometric mean of virus titers measured during the entire experimental period), (2) peak virus shedding titer and (3) duration of virus shedding in days. To combine and compare virus shedding levels across studies, only studies that reported virus shedding levels in either EID_50_ (virus isolation) or EID_50_-equivalents (real time-PCR) were assessed. Studies that reported outcomes in threshold cycle (ct), TCID_50_, PFU and ELD_50_ were not included in the meta-analysis. Studies from which data from individual animals could not be extracted were also not included. Reported data of virus isolation and real time-PCR results were first assessed for similarity and were found to be highly similar [25]. Virus shedding levels were transformed to log_10_ EID_50_ to minimize variance between studies. Geometric means of virus shedding levels were calculated for every observation.

Generalized linear multivariable mixed models (GLMM) were used to model the association between virus shedding levels (geometric mean or peak shedding) and the variables that could have an effect on shedding patterns (explanatory variables). In these models, peak or geometric mean shedding were the response variables. Virus subtype, inoculation route, poultry species, etc. and possible interactions between these variables were included as explanatory variables (see Table 1 for a description of these variables). To account for variation between studies, study was introduced as a random effect in the model. Initial data exploration showed that the pathotype of the virus (HPAIV or LPAIV) modified the effect of several variables including subtype and species. Therefore, one model was fit to compare shedding between HPAIV and LPAIV infections and then separate models were fitted for HPAIV and LPAIV. It was also observed that studies that used an intranasal inoculation route or combination of other inoculation routes (intratracheal and oropharyngeal) together with intranasal inoculation showed similar virus shedding levels. To reduce the number of variables assessed in the models, intranasal inoculation routes were pooled. The age when poultry becomes reproductive defines the distinction between young and old poultry. For chickens and turkeys this age was set at 126 days (18 weeks) and for all other poultry species this was set up at 150 days. Backward selection was used to build the models, the significance level was set to *p* < 0.05, with significant variables and variables showing a confounding effect being kept in the final models. Variables considered biologically relevant were also kept in the model, regardless of their *p*-value. Goodness of fit of the models was assessed by inspecting residuals.

Shedding duration was assessed by fitting parametric survival models. All fixed effects that were evaluated for their effect on virus shedding levels, were also evaluated in the survival analysis. The lognormal or Weibull distributions fitted best the data. Weibull models showed the best fit for modeling the length of LPAIV shedding and lognormal models had the best fit for HPAIV. Selection of the distribution used in the parametric models was based on (1) the model fit, assessed by evaluating the agreement between the fitted survival curve and the non-parametric (Kaplan Meier) survival estimates and (2) by competing different parametric models (models using different distributions) using the Akaike information criterion (AIC) and choosing the models with the lowest AIC. Similar to the model of virus shedding levels, a backwards elimination procedure was used. Only data where the complete shedding period was observed (followed up daily or every two days) or where data was right censored (last test positive before the experiment was terminated) were included. For contact infected animals, it was assumed that they were infected the day before they tested first positive [26,27] in either respiratory or cloaca samples. Experiments in which the first day of sampling was ≥3 day post inoculation (dpi) were also excluded from this analysis. In total 592 (334 censored) observations were included for analysis of LPAIV virus shedding duration and 451 (127 censored) were included for analysis of HPAIV virus shedding duration. For shedding duration estimations of LPAIV only non-censored and interval censored data could be used, as including right censored data led to a lack of model fit. Statistical analysis were done using the software R 3.4.0 [28], GLMM were fitted using the package lme4 [29] and survival models using the package survival [30].

## 3. Results

### 3.1. Literature Search and Screening

**Figure 1:**
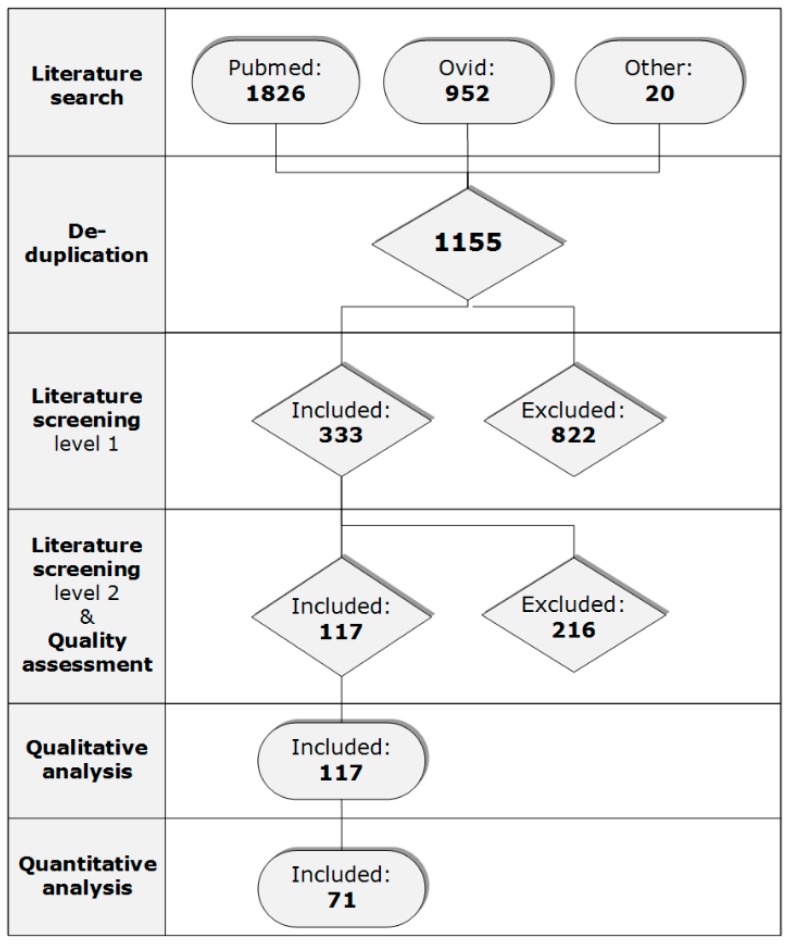
The number of the included and excluded citations during the literature search, both literature screening levels, quality assessment and both analyses.

The last search in the electronic databases was performed on 19th July 2017. During the literature search, 2798 citations were retrieved in total. The database PubMed provided 1826 citations and the search in Ovid, with searching in three databases simultaneously, resulted in 952 citations (Figure 1). Twenty citations or data sources were added to the database by other ways than searching in databases with the determined search terms, i.e., identifying by hand searching or receiving grey data. After deduplication, 1155 citations remained for the first literature screening. During the screening, 822 studies were discarded and 333 publications remained based on their title and abstract. The most common reason for excluding was the use of another experimental unit than poultry. Full texts of the remaining studies were examined on the selection criteria of the second screening, after which 115 studies and two unpublished databases provided by R. Maas and J. L Gonzales, both working at WBVR, remained for the qualitative analysis. During the second screening, most common reasons for exclusion were failure of reporting quantitative virus shedding patterns, irrelevance and missing data. Some manuscripts were excluded during quality assessment for not using one of the selected outcomes, reporting shedding values without units or the reported units were unclear.

### 3.2. Data Analysis and Summation

#### 3.2.1. Qualitative Analysis

**Figure 2:**
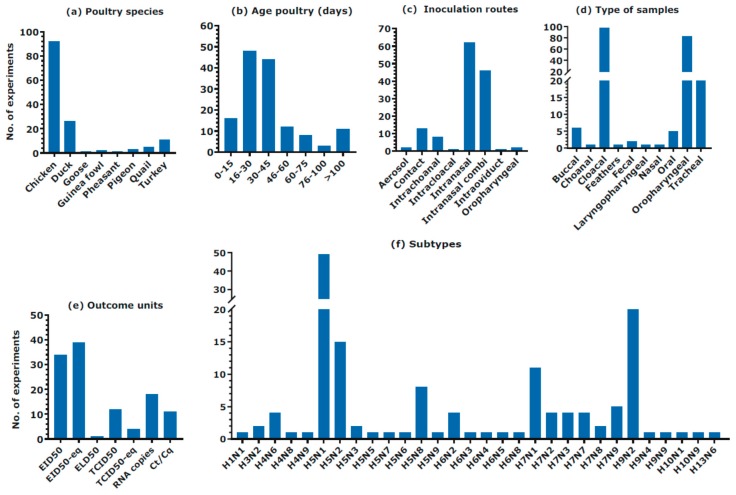
An overview of the qualitative analysis. The different studied poultry species, ages, inoculation routes, types of samples, outcome units and virus subtypes in the included studies. Some studies used multiple variables. “Intranasal combi” is a collective term for all different intranasal combinations e.g., intranasal and ocular inoculation.

The qualitative analysis was performed for 117 studies. Table S3 is provided as a Supplemental Materials and presents all included studies. The study population consisted of 880 experimental units in total, including chicken, duck, goose, guinea fowl, pheasant, pigeon, quail and turkey. Figure 2 presents all studied poultry species, ages, inoculation routes, type of samples, outcome units and subtypes of the included studies. Chickens were studied in 92 of the 117 studies, which made them the most studied poultry species. Ducks and turkeys were studied in 26 and 11 studies, respectively. The age of the infected animals varied from 5 to 994 days, but most experiments used 16- to 45-day-old poultry. A large variation existed between the used inoculation routes, with some variation explained by the different aims of the studies, e.g., in one study, the intra-oviduct inoculation route was chosen to assess this route as a potential infection route in turkeys. The intranasal inoculation route, or a combination of intranasal with diverse respiratory inoculation routes, was the most often used method for inoculation. Thirteen studies used contact infected poultry. In total, 10 different types of samples were collected in the included experiments. Oropharyngeal and cloacal swabs were most often used for sampling virus shedding, 83 and 97 times respectively. In the included studies, seven different outcome units were used of which the EID_50_ and EID_50_-equivalent were the most used units to describe viral shedding. Overall, 30 different subtypes were examined where H5N1 was the most studied subtype (*n* = 49). H5N1 represents the most studied HPAIV, whereas H9N2 was the most studied LPAIV.

#### 3.2.2. Quantitative Analysis (Meta-Analysis)

**Figure 3:**
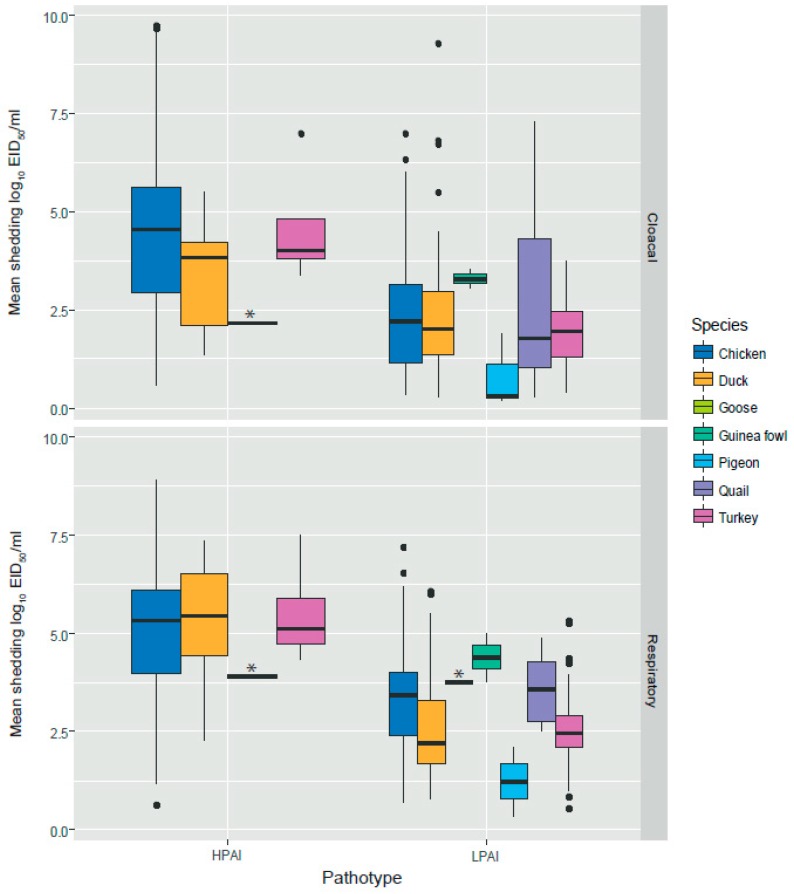
Distribution of mean shedding levels (log_10_ EID_50_/mL) in respiratory and cloacal swabs of highly pathogenic avian influenza (HPAI) virus and low pathogenic avian influenza (LPAI) virus distributed by species. The box represents the first quartile, the median (band inside the box) and the third quartile. Individual black dots are outliers. * indicates goose data (box is not visible because there was only one observation).

Data from 71 studies were used for the meta-analysis (Table S3). These data provided information on the mean and peak virus shedding level (log_10_ EID_50_/mL) and duration of shedding (days). Combined data from these studies resulted in a total of 322 and 381 experimental animals, infected with HPAIV and LPAIV respectively, providing information for the analysis. Table 1 presents an overview of the analyzed data and an overview of the mean respiratory and cloacal shedding levels for HPAIV and LPAIV in different species is given in Figure 3. This figure shows the variation in shedding levels and the information available for different poultry species, e.g., limited information is available for geese.

#### 3.2.3. Shedding Levels

**Figure 4:**
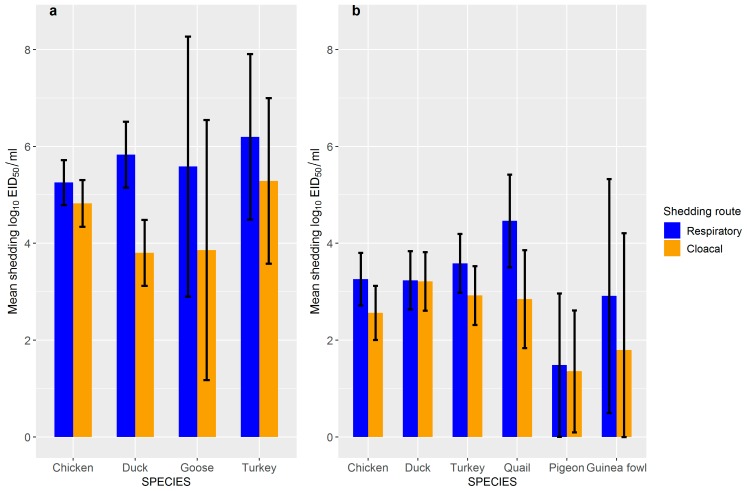
Mean shedding (log_10_ EID_50_/mL) in respiratory and cloacal swabs of (**a**) highly pathogenic avian influenza virus (HPAIV) and (**b**) low pathogenic AIV (LPAIV) in chicken, duck, goose, turkey, quail, pigeon and guinea fowl after correcting for the effect of virus subtype, inoculation route, dose, virus origin, etc. Error bars represent 95% confidence intervals for the mean estimates.

Statistical analyses were done fitting GLMM, which was allowed to make corrections for the effect of virus subtype, inoculation route, dose, virus origin, etc. when comparing shedding between different species. First, levels of shedding between poultry infected with LPAIV and HPAIV were compared. This analysis showed that overall shedding (mean and peak) was significantly lower (*p* < 0.001) in poultry (chickens, ducks and turkeys) infected with LPAIV than HPAIV, with an estimated average difference in mean and peak shedding of 1.82 and 3.14 log_10_ EID_50_/mL of the sample, respectively (Figure 4, Table 2).

Second, we fitted GLMM to assess shedding of HPAIV and LPAIV separately. Table 2 presents the results of the analysis done for HPAIV shedding in detail and adjusted estimates of mean shedding for all species studied are shown in Figure 4a. It was observed that respiratory shedding levels are higher than cloacal shedding levels for all species (*p* < 0.001). Ducks shed via the respiratory route higher amounts of virus (*p* < 0.001) and via the cloacal route lower amounts of virus than chickens (*p* = 0.008; Table 2, Figure 4a). In chickens, the amount of virus shedding is lower when the virus, used for inoculation, originates (was isolated) from birds from a different species (from the same or a different order) than when the virus originates from chickens (same species; *p* < 0.001). This association was not found in ducks, and available data did not allow us to make this assessment for turkeys. Age had a significant effect on shedding, with young poultry shedding less of the virus than adult poultry (*p* < 0.001). There were no significant associations found for the inoculation routes (all species) or the type of chickens (broiler vs. layers) with shedding levels. Differences were also observed between virus subtypes, with differences observed between viruses having different hemagglutinin (H5 or H7) and between viruses having the same hemagglutinin (e.g., between H5 viruses; Figure S1a). To simplify the analysis, and due to data limitations, we did not draw independent interpretations per subtype and we limited analysis to include subtype in the models to adjust for its effect when assessing the effect of the other variables of interest.

Table 3 presents the variables affecting shedding levels in poultry infected with LPAIV and adjusted estimates of mean shedding for each of the poultry species studied are shown in Figure 4b. For LPAIV, the level of virus that was shed in respiratory samples was, like HPAIV, higher than the level of virus that was shed by the cloaca for all species except ducks and pigeons. When the level of shedding of different species were compared with chickens, it was found that ducks and turkeys shed higher virus levels via the cloaca than chickens (*p* < 0.001), whilst quails had a higher respiratory shedding level (*p* = 0.007). Pigeons had lower respiratory and cloacal shedding levels than chickens (*p* = 0.042). Virus origin had the same effect on shedding for LPAIV as observed for HPAIV, with chickens infected with virus isolated from chickens shedding higher levels of virus (*p* < 0.01). In contrast to HPAIV, there was a positive association between the inoculation route and shedding of LPAIV, with aerosol (only chickens), intranasal and oropharyngeal inoculated poultry shedding higher levels of virus than contact infected poultry. There were no significant differences in shedding levels between other inoculation routes and contact infected poultry or age and type of chickens for LPAIV. As for HPAIV, virus subtype had a significant effect on shedding (Figure S1b) and this variable was used in the models to adjust for the effect of the other assessed variables.

**Figure 5:**
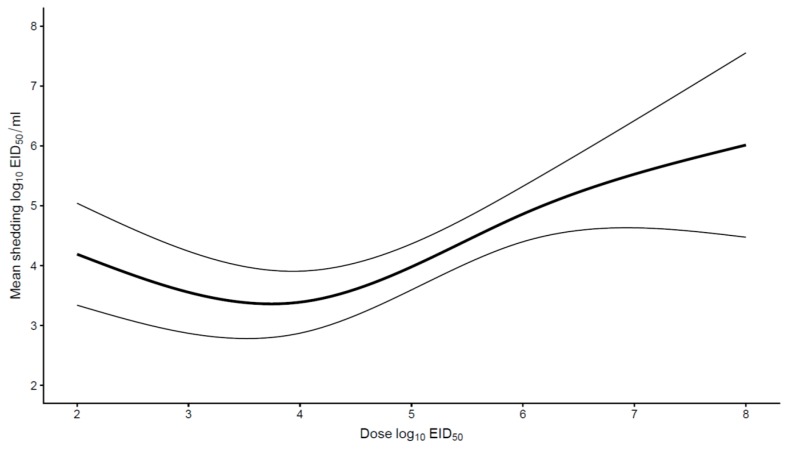
The relationship between the inoculation dose and the mean respiratory and cloacal virus shedding (log_10_ EID_50_/mL) for HPAIV in chickens. The mean shedding increased when poultry were inoculated with doses above 10^5^ EID_50_.

Finally, an analysis was performed to search for a relationship between the inoculation dose and virus shedding. Most studies involving poultry species other than chickens used similar doses (ranging from 10^5^ to 10^6^ EID_50_), hence it was only possible to assess this relationship only for chickens. For HPAIV infections, doses used ranged from 10^2^ to 10^8^ EID_50_. There was a positive association between inoculation dose and shedding when doses were above 10^5^ EID_50_, the higher the dose, the higher the shedding (Figure 5). In contrast, no significant association between dose and shedding was found for LPAIV. Inoculation doses ranged from 10^2^ to 10^9.6^ EID_50_.

#### 3.2.4. Duration of Shedding

Since data was frequently censored, it was not possible to make a detailed assessment of the duration of virus shedding as was done for the mean and peak of shedding. Survival models with many variables did not produce a good fit and therefore the assessment was limited to an overall comparison of duration of shedding between species. An indication of the differences in duration of respiratory and cloacal AIV shedding between different poultry species is given in Table 4. Ducks shed HPAIV via the respiratory or cloacal route around four days longer than chickens (*p* < 0.01). In both species the shedding duration of HPAIV via the respiratory route is similar to that via the cloacal route. LPAIV behaved differently from HPAIV: The duration of respiratory virus shedding in ducks was not different to that in chickens (*p* > 0.05) but the duration of cloacal shedding appeared to be longer in ducks than in chickens (*p* = 0.08). As for turkeys, cloacal shedding appeared to be longer than chickens (*p* = 0.05). There were no significant differences in the duration of virus shedding via both respiratory or cloaca routes for other species. However, data were limited, thus limiting power, and resulting in large confidence intervals for the estimates (Table 4).

## 4. Discussion

The main objective of this review was to collect and summarize quantitative information on virus shedding levels and the duration of shedding of different AIV subtypes by different poultry species. It became clear that a large heterogeneity in experimental methods exists between studies, illustrated by the use of eight different combinations of inoculation routes, ten different sample sites and seven different ways of measuring/reporting shedding. An important motivation for this systematic review was therefore to identify the effects of differences in experimental methods on shedding levels. In addition, the quantitative analysis allowed assessing differences in shedding among different poultry species. The analysis showed for example that HPAIV shedding was higher than LPAIV shedding in all poultry species and that the virus origin (host from which virus was primarily isolated) had a significant influence on shedding of both HPAIV and LPAIV only for chickens. It was also observed that the inoculation route had no significant influence on shedding of HPAIV whilst the inoculation route did have an influence on shedding of LPAIV.

With many AIV subtypes and different poultry species being at risk, challenge studies contribute to improving our understanding of AIV infections in poultry by providing observations that taken together could lead to robust identification of risk factors associated with infection. However, our review highlights an observed large heterogeneity in experimental methods and outcome units used, limiting comparability of experimental results and therefore the confidence in conclusions drawn when considering the evidence of similar experiments together. In detail, 46 out of 117 studies selected for the qualitative analysis could not be included in the quantitative analysis, hence limiting the power of our quantitative analysis and the range of variables that could be assessed (e.g., subtype specific assessments). Some level of standardization, such as reporting shedding levels in say EID_50_ or EID_50_ equivalents (the most common outcome units observed in this review), would improve comparability of the studies. We would encourage experimentalists to strive for standardization of the experimental method, although we acknowledge that the experimental methods sometimes must vary due the objective of the study, e.g., to identify infection routes [12,31,32]. In addition, it should be noted that quality of reporting compromised the use of many studies, with 65% (216 studies out of 333) of selected studies in the first literature screening not passing the second literature screening and quality assessment, restricting therefore the use of the majority of the studies for this review and the power of the quantitative analysis. It is clear that the quality of reporting in AIV challenge experiments needs to be improved. Following guidelines such as the ARRIVE (Animal Research: Reporting of In Vivo Experiments) guidelines [33] to report animal experiments would help improve reporting.

One of the variables that was analyzed in the quantitative analysis was the pathogenicity of the virus. It was shown that poultry shed more of the virus after inoculation with HPAIV compared to LPAIV. This high HPAIV shedding caused a high viral load in the environment, which could make indirect virus transmission between flocks or farms more effective, due to a higher chance for virus contamination of materials, for example, trucks or boots. The higher virus shedding by infection with HPAIV could be an explanation for the enhanced transmissibility of HPAIV H7N1 compared to the LPAIV variant as described in [34].

In addition to the pathogenicity of the virus, the effect of experimental methods, such as inoculation dose, on shedding was included in the quantitative analysis. The inoculation dose could only be assessed for chickens. For HPAIV it appears that using inoculation doses above 10^5^ EID_50_ results in a linear increase in virus shedding. Spekreijse et al. [35] previously studied the effect of inoculation dose on transmission of HPAIV H5N1 from inoculated chickens to contact chickens. It was shown that increasing the dose significantly increased the amount of virus shed from the trachea and cloaca in the time between inoculation until contact infection with a native pen mate and shortened mean latent period. On the other hand, the transmission rate parameter and reproduction ratio were not significantly affected by the inoculation dose. Similar observations were reported by Bouma et al. [27]. Based on these results and the observation in the quantitative analysis, for HPAIV a maximum inoculation dose of 10^5^ EID_50_ could be used in future challenge studies to infect all experimental units but to avoid affecting the mean virus shedding level.

In contrast to HPAIV, no significant association between the inoculation dose and LPAIV shedding was found. It might be expected that for LPAIV the inoculation dose is more critical compared to HPAIV as it is less virulent and pathogenic, a relatively low dose of HPAIV might be sufficient for infection, whilst infection with LPAIV requires a higher dose to achieve virus shedding in chickens [36,37]. At lower doses not all experimental units might be infected, which cannot be seen from the data as only poultry that shed viruses were included in the meta-analysis. Given the low virulence and pathogenicity of LPAIV, it could be that once chickens become successfully infected with LPAIV, regardless of the inoculation dose, they would end up shedding similar levels of virus. Considering the need of ensuring that most (or all) poultry become infected during performing experiments and the observation that there is no effect of the inoculation dose on LPAIV shedding, high doses (around 10^6^ EID_50_/mL [36,37]) may be advisable for LPAIV experiments.

As for the inoculation route, no differences in shedding were found between the intranasal or intrachoanal routes of inoculation and contact infected poultry for HPAIV. These findings appear to be in line with the study by Pantin-Jackwood et al. [32], which was included in the quantitative analysis, in which it was shown that intranasal, intracloacal and intraocular inoculation routes results in similar virus shedding patterns. In contrast to HPAIV, an association was found for LPAIV shedding, with aerosol, intranasal and oropharyngeal inoculation leading to higher shedding levels than contact infection. It has been suggested that for LPAIV experimental inoculation routes might favor localized shedding, for example intranasal and intratracheal inoculation may favor localized respiratory infection [38]. However, we could not make a formal assessment of the interaction between inoculation route, sampling site and subtype because of data limitations. As for the contrasting observations between HPAIV and LPAIV, it could be speculated that due to the high pathogenicity and virulence of HPAIV the inoculation route is of less influence compared to LPAIV. For LPAIV, as well as for HPAIV, no differences were found for intrachoanal inoculation and contact infection, hence intrachoanal inoculation may be the inoculation route that best mimics shedding following natural infection (contact infection).

In addition to the expected differences in virus shedding between various experimental methods, it was expected that virus shedding would differ among poultry species in both shedding route and shedding levels. Traditionally it is thought that viral replication of AIV in ducks occurs primarily in epithelial cells of the intestinal tract, and high concentrations of virus are shed in the feces, whereas virus replication is primarily respiratory in chickens [39,40,41,42]. Our results, after correcting for subtype and other experimental variables provide more accurate insights. For HPAIV, respiratory shedding is higher than cloacal shedding for all poultry species and ducks shed even higher levels (for a longer period) of virus than chicken via the respiratory tract and lower levels of virus than chickens via the cloaca. As for LPAIV infections, ducks shed (mean and peak shedding) similar levels via the respiratory as the digestive tract. On the other hand, ducks shed higher levels of virus (and for a longer period) via the cloaca than chickens.

According to the analysis, turkeys shed also higher levels of LPAIV via the cloaca than chickens. Due to this higher level of virus shedding, more of the virus will be present in the environment of turkeys (e.g., bedding materials and feathers) and therefore the virus transmission is probably higher. The higher viral load in the environment of turkeys, in combination with the higher susceptibility of turkeys for LPAIV [43,44], and the long period of shedding (see “Duration of shedding”), increases the chance that LPAIV spread successfully in a turkey flock compared to a chicken flock. This was also shown by calculations of the basic reproduction ratios (R_0_) for a LPAIV H7N1 virus in both species: The R_0_ in turkeys was 15.3 (11.8–19.7) [45] whilst in chickens the R_0_ was 3.8 (1.3–6.3) [34]. The increased risk of LPAIV transmission in turkeys could be further supported by the absence of statistical association between virus origin and virus shedding by turkeys. Contrary to turkeys, virus origin has a significant effect on virus shedding by chickens. If a virus (HPAIV or LPAIV) originates from the same species (chickens), it results in higher virus shedding levels than when the virus originates from another host species from the same (e.g., turkeys) or a different (e.g., wild ducks) order. This may have implications for transmission, because when the virus originates from a different order (e.g., wild ducks) there is a good chance that the infection will die out within a chicken flock because of the low virus shedding by chickens. However, after a virus introduction into a turkey flock, turkeys will shed high amounts of virus, even when the virus originates from wild birds.

Data for the variable “age” was difficult to obtain for all poultry species and ages, hence our age findings were mainly applicable to young and adult chickens. Virus shedding was expected to decrease with age (except for very old poultry), as animals become fully immunocompetent and viral pathogenicity in immunocompetent hosts is decreased. However, we observed that young (non-reproductive) animals shed significantly lower amounts of HPAIV than adults (reproductive). A study here reviewed [9] showed that in addition to virus shedding, the mean death time, mean bird infectious (BID_50_) and lethal (BLD_50_) doses were not significantly different for broilers of five, eight and 30 weeks after inoculation with a HPAIV H5N2. Thus, for this virus there was neither a difference in susceptibility nor virus shedding between the different age groups of chickens. Most of the data for this assessment were for chickens with a limited number of observations coming from other species, hence these results were mainly representative for chickens and additional research (data) is needed to be able to confirm this observation for other poultry species. For LPAIV no significant differences in virus shedding were found between young and adult chickens (this assessment was done only for chickens). The finding for LPAIV in chickens was comparable with the results of the study of Lavoie [46], in which the effect of ages on immunologic responses and LPAIV shedding was investigated in different ages of quails (two weeks, six weeks, 10 months and 28 months old), which were inoculated with a H9N2 virus. No significant association in virus shedding between the different age groups was found, even though differences were seen in the immunologic responses. The highest T-cell associated immune response and humoral response in the blood were detected in the 10-month-old-quails. The 28-month-old-quails had the lowest immune and humoral response and the clinical symptoms were also worst in this group. Based on the results of the quantitative analysis and Lavoie, it appears that age is not associated with LPAIV virus shedding in chickens and quails. However, VanDalen [47] detected lower shedding levels of LPAIV in three month-old-mallards than in six-month-old mallards and Costa [48] detected lower shedding levels of LPAIV in two-week-old mallards than in mallards of at least one month old. A possible explanation for the lower LPAIV shedding in non-reproductive mallards may be that reproductive mallards spend more energy on reproduction and less on immunity. Further research could provide a more definitive answer.

The authors were aware of limitations and some potential sources of bias in this review, which must be taken into account when drawing conclusions. During summarizing and analyzing data, the observations of the same subtype, regardless of their origin, were pooled. Combining these observations was a potential source of bias. Differences in virus shedding patterns might exist between different strains of the same subtype, which could be the case for example for the HPAIV H5N1 strains [19]. Hence the conclusion might be generic and the existence of virus strains showing some different behaviors to that reported in our results cannot be excluded. Another potential source of bias was the selection procedure of studies, due to resource limitations, papers were selected by only one reviewer. Ideally papers are selected by at least two reviewers, limiting the risk of selection bias. Hence a strict SR plan and selection criteria were followed in order to minimize this risk. Housing of experimental animals might be also a source of bias. It was not clear for many experiments whether inoculated animals were housed individually or kept as a group. If the latter was the case the assumption had to be made that inoculation was 100% effective. If inoculation failed to infect some animals and they became infected via contact, then comparisons between inoculation route and contact-infection need to be carefully considered. Finally, data availability and the large heterogeneity in experimental methods limited this assessment, this was particularly the case for the assessment of the duration of shedding and/or assessment, for example, of the effect of interactions between the inoculation route, sampling site and subtype on shedding.

To conclude, this review showed the large heterogeneity in the experimental methods of challenge studies. More standardization in the experimental methods and reporting, for example reporting the outcome unit in EID_50_ or EID_50_ equivalents, will improve the comparability of individual studies. When studies are more comparable, the formal assessments for variables that could not be performed during this meta-analysis because of data limitations could be performed in the future. Using guidelines for reporting animal experiments, such as the ARRIVE guidelines, will improve the quality of reporting. The quantitative analysis showed that some experimental variables affect the virus shedding patterns for HPAIV, LPAIV or both. For this reason, all variables should be considered carefully before starting a challenge experiment and here we identified some variables that could lead to some level of standardization (e.g., inoculation route). This review could be used as a background for the design of future challenge studies for AIV.

## Figures and Tables

**Table 1:** Available data extracted from the included studies (*n* = 71) used for the meta-analysis.

Explanatory Variables	Levels	Number of Observations		Percentage of Observations (%)	
		HPAIV	LPAIV	HPAIV	LPAIV
Species The poultry species that were infected	Chicken	484	477	86.43	65.3
	Duck	66	112	11.79	15.3
	Turkey	8	108	1.43	14.8
	Goose	2	2	0.36	0.3
	Pigeon		8		1.1
	Quail		16		2.2
	Guinea fowl		4		0.5
	Pheasant		3		0.4
Age ^a^ The age at infection	Adult	70	109	12.50	14.9
	Young	490	621	87.50	85.1
Inoculation route Site at which the animal is experimentally inoculated with AIV	Aerosol		4		0.5
	Contact	17	231	3.04	31.6
	Intrachoanal	119	191	21.25	26.2
	Intracloacal		4		0.5
	Intranasal (combi)	424	276	75.71	37.8
	Intraoviduct		4		0.5
	Oropharyngeal		20		2.7
Subtype Subtype of the inoculated virus	H3N2		4		0.5
	H5N1	349	11	62.77	1.5
	H5N2	165	239	29.68	32.7
	H5N3		42		5.8
	H5N5		11		1.5
	H5N6	4			0.8
	H5N7		6		
	H5N8	16	9	2.88	1.2
	H5N9		11		1.5
	H6N2		38		5.2
	H7N1	16	104	2.88	14.2
	H7N2	2	54	0.36	7.4
	H7N3	6	18	1.08	2.5
	H7N7	2	72	0.36	9.9
	H7N8		6		0.8
	H7N9		64		8.8
	H9N2		38		5.2
	H10N9		3		0.4
Virus origin Species from which the virus originates	Same species	146	392	26.07	53.7
	Same phylogenetic order	333	95	59.46	13.0
	Different phylogenetic order	81	225	14.46	30.8
	Origin unknown		18		2.5
Sample site Body part where a sample was taken for determination of the virus shedding levels	Buccal	5	4	0.89	0.5
	Cloacal	238	357	42.50	48.9
	Tracheal	91	181	16.25	24.8
	Oropharyngeal	183	187	32.68	25.6
	Nasal	1	1	0.18	0.1
	Oral	38		6.79	
	Laryngopharyngeal	4		0.71	
Type	Layer	418	177	86.36	37.11
Production type of commercial chickens	Broiler	28	3	5.79	0.63
	Unknown	38	297	7.85	62.26

The number of observations was equal to the amount of samples. Empty cells: Data not available; HPAI: Highly pathogenic avian influenza; LPAI: Low pathogenic avian influenza; ^a^ the age when poultry becomes reproductive defines the distinction between young and old poultry. For chickens and turkeys this age was set at 126 days (18 weeks) and for all other poultry species this was set up at 150 days.

**Table 2:** Variables affecting shedding (assessed as geometric mean shedding and peak shedding) level in chicken, duck, turkey and goose infected with HPAIV.

Explanatory Variables	Poultry Species	Effect	Difference in Mean Shedding (log_10_ EID_50_/mL) ^a^	Difference in Peak Shedding (log_10_ EID_50_/mL) ^a^	p (Mean|Peak)
Pathotype (HPAI vs. LPAI)	All	Poultry infected with LPAI shed lower levels of virus than poultry infected with HPAI	−1.82 ± 0.33	−3.14 ± 0.51	<0.001|<0.001
Shedding route	All	Respiratory shedding is higher than cloacal shedding for all species	0.43 ± 0.12	0.42 ± 0.15	<0.001|0.005
	Turkeyand goose	NS differences between turkeys and geese with chickens	NS	NS	>0.05|>0.05
	Duck	Higher respiratory shedding than chickens	0.58 ± 0.35	0.75 ± 0.39	<0.001|<0.001
		Lower cloacal shedding than chickens	−1.02 ± 0.38	−0.93 ± 0.43	0.008|0.03
		Higher respiratory shedding than cloacal	1.96 ± 0.17	2.10 ± 0.31	<0.001|<0.001
Virus origin	Chicken	Shedding is lower when virus originates from birds from the same order than when the virus originates from the same species	−0.89 ± 0.25	−0.62 ± 0.30	<0.001|0.04
		Shedding is lower when virus originates from birds from different orders than when the virus originates from the same species	−0.92 ± 0.29	−0.93 ± 0.34	<0.002|0.007
	Duck	NS differences in shedding between the origin of the virus	NS	NS	>0.05|>0.05
Inoculation route	All	NS differences in shedding between intranasal and intrachoanal routes versus contact infected poultry ^b^	NS	NS	>0.05|>0.05
Age	All	Young poultry shed lower than adults ^c,d^	−1.07 ± 0.21	−1.03 ± 0.25	<0.001|<0.001
Type	Chicken	NS differences in shedding between broilers and layers	NS	NS	>0.05|>0.05

NS = not significant; ^a^ the observed differences in mean or peak shedding (in log_10_) for all subtypes in general. For example: In general, the mean virus shedding in poultry after inoculation of HPAIV is on average almost 2 log_10_ higher than for LPAIV. ^b^ Inoculation lead to slightly higher shedding levels (NS) than contact infections. ^c^ Mainly chicken data. ^d^ In ducks only young animals were assessed.

**Table 3:** Variables affecting shedding (assessed as geometric mean shedding and peak shedding) levels in poultry infected with LPAIV.

Explanatory Variables	Poultry Species	Effect	Difference in Mean Shedding (log_10_ EID_50_/mL)	Difference in Peak Shedding (log_10_ EID_50_/mL)	p (Mean|Peak)
Shedding route	All	Respiratory shedding is higher than cloacal shedding with the exception of ducks and pigeons	0.70 ± 0.11	2.15 ± 0.14	<0.001|<0.001
	Duck	Higher cloacal shedding than chickens	0.65 ± 0.17	1.19 ± 0.27	<0.001|<0.001
		NS difference between cloacal and respiratory shedding	NS	NS	>0.05|>0.05
	Turkey	Higher cloacal shedding than chickens	0.36 ± 0.17	1.16 ± 0.27	<0.001|<0.001
	Pigeon	Lower respiratory and cloacal shedding than chickens	−1.21 ± 0.59	−2.42 ± 0.83	0.042|0.003
	Quail	Higher respiratory shedding than chickens	1.19 ± 0.44	2.42 ± 0.65	0.007|<0.001
	Guinea fowl	NS differences in shedding between chickens and guinea fowl	NS	NS	>0.05|>0.05
Virus origin	Chicken	Shedding is lower when the virus originates from birds from the same order than when the virus originates from the same species	−1.17 ± 0.28	−1.35 ± 0.78	<0.001|0.01
		Shedding is lower when the virus originates from birds from different orders than when the virus originates from the same species	−0.73 ± 0.27	−2.08 ± 0.67	0.007|0.005
	Duck	NS differences in shedding between the origin of the virus	NS	NS	>0.05|>0.05
	Turkey	NS differences in shedding between the origin of the virus	NS	NS	>0.05|>0.05
Inoculation route	Chicken	Aerosol inoculated chickens shed higher than contact infected chickens	2.04 ± 0.66	2.72 ± 1.04	0.002|0.009
	All	Intranasal inoculated poultry shed higher than contact infected poultry	0.61 ± 0.14	0.77 ± 0.71	<0.001|<0.001
	All	Oropharyngeal inoculated poultry shed higher than contact infected poultry	2.44 ± 1.13	2.66 ± 1.13	0.042|0.03
	All	NS differences in shedding between intrachoanal, intracloacal and intra-oviduct inoculation routes and contact infected poultry	NS	NS	>0.05|>0.05
Age	Chicken	NS differences in shedding between ages ^a^	NS	NS	>0.05|>0.05
Type	Chicken	NS differences in shedding between broilers and layers ^b^	NS	NS	>0.05|>0.05

NS = not significant; ^a^ in ducks and turkeys only young animals were assessed. ^b^ For broilers there were only three observations available.

**Table 4:** An indication of AIV shedding length in days in multiple poultry species.

Pathotype	Poultry Species	Length of Respiratory Virus Shedding (Days)		Length of Cloacal Virus Shedding (Days)	
HPAI	Chicken	2.6	(1.1–6.5)	2.5	(1.0–6.2)
	Duck	6.9	(2.8–17.1)	6.6	(2.7–16.3)
LPAI	Chicken	6.2	(0.8–17.8)	5.5	(0.7–15.7)
	Duck	5.3	(0.7–15.3)	8.2	(1.0–23.3)
	Turkey	10.0	(1.3–28.7)	14.1	(1.8–40.2)
	Guinea fowl	3.3	(0.4–9.4)	3.3	(0.4–9.4)
	Pigeon	3.6	(0.4–10.2)	2.8	(0.3–8.0)
	Quail	NA		6.9	(0.9–19.8)

NA = not applicable.

## Supplementary Materials

The following are available online at https://www.mdpi.com/1999-4915/11/9/812/s1, Figure S1. Mean shedding (log_10_ EID_50_/mL) in respiratory and cloacal swabs of a) HPAIV and b) LPAIV for different subtypes. Table S1. Summary of the systematic review protocol, Table S2. Complete overview of search terms used in PubMed and Ovid, Table S3. References of all included studies.
