# Supplementary material for: Virus Shedding of Avian Influenza in Poultry: A Systematic Review and Meta-Analysis
Source: Viruses. 2019 Sep 2;11(9):812. doi: 10.3390/v11090812 (PMC6784017; doi:10.3390/v11090812)
Supplement: Supplementary file 1 [file viruses-11-00812-s001.zip › AI_Shedding_Suppl_Tables.docx]

**Supplementary table 1.** Summary of the systematic review protocol.

| Review question | Which characteristics influence the level (mean and peak) and length of AIV shedding in experimentally infected poultry? |
| --- | --- |
| Search date | Until 19 July 2017 |
| Language | English |
| Publication types | - Peer reviewed papers:   - Primary research were AIV shedding is quantified   - The challenge control group of AIV vaccination studies can also provide data - Grey literature: unpublished experiments about AI virus shedding |
| Population | Any species or breed of poultry of all ages will be included in the literature search.  “Poultry” is defined as: all birds that are reared or kept in captivity for the production of meat or eggs for consumption, the production of other products, for restocking supplies of game birds or for the purposes of any breeding programme for the production of these categories of birds, including chickens, duck quail, goose, turkey, pheasant, guinea fowl, partridge, swans, ostrich and pigeon. |
| AI strains | HPAI and LPAI viruses of all subtypes |
| Inoculation routes | All routes and combinations |
| Outcome | Quantified virus shedding and/or length.  Allowed outcome units:   - 50% egg infectious dose (EID_50_) and EID_50-equivalents_ - 50% egg lethal dose (ELD_50_) - 50% tissue culture infective dose (TCID_50_) and TCID_50-equivalents_ - RNA/PCR copies - thresholds cycle (C_t_), quantification cycle (C_q_) or crossing point (C_p_) |

**Supplementary table 2.** Complete overview of search terms used in Pubmed and Ovid.

| **Database** | **Search term** | **Hits^a^** |
| --- | --- | --- |
| Pubmed | ("Influenza in Birds"[Mesh]) AND "Virus Shedding"[Mesh] | 201 |
|  | (("Influenza A virus"[Mesh]) AND "Poultry"[Mesh]) AND "Virus Shedding"[Mesh] | 160 |
|  | avian influenza poultry shedding | 369 |
|  | avian influenza poultry pathology | 429 |
|  | avian influenza poultry secretion | 36 |
|  | avian influenza poultry excretion | 43 |
|  | ("Influenza in Birds"[Mesh]) AND "Disease Transmission, Infectious"[Mesh] | 119 |
|  | avian influenza transmission quantification | 16 |
|  | avian influenza virus titres poultry | 78 |
|  | (H5N1 OR H5N2 OR H5N7 OR H5N8 OR H5N9 OR H7N1 OR H7N7 OR H7N9 OR H9N2) AND virus shedding AND poultry | 360 |
|  | avian influenza transmission parameters | 63 |
|  | ("Influenza A virus"[Mesh]) AND ( "Intestinal Elimination"[Mesh] OR "Pulmonary Elimination"[Mesh] OR "Salivary Elimination"[Mesh] ) | 0 |
|  | Influenza in Birds[Mesh] AND ( "Intestinal Elimination"[Mesh] OR "Pulmonary Elimination"[Mesh] OR "Salivary Elimination"[Mesh] ) | 0 |
|  | ("Influenza in Birds"[Mesh]) AND "Bodily Secretions"[Mesh] | 4 |
|  | ("Influenza A virus"[Mesh]) AND "Bodily Secretions"[Mesh] "Poultry"[Mesh]) | 7 |
|  | avian influenza poultry transmission parameters | 38 |
| Ovid | avian influenza and poultry and shedding | 618 |
|  | avian influenza and poultry and excretion | 100 |
|  | avian influenza and poultry and secretion | 39 |
|  | avian influenza and transmission and quantification | 33 |
|  | avian influenza and poultry and transmission parameters | 16 |
|  | (H5N1 or H5N2 or H5N7 or H5N9 or H7N1 or H7N7 or H7N9 or H9N2) and virus shedding and poultry | 218 |
|  | avian influenza and poultry and virus titres | 27 |

^a^ number of hits before deduplication.

**Supplementary table 3.** References of all included studies.

| **References of included studies (n=117)** | **Used for meta-analysis (n=71)** |
| --- | --- |
| **Barjesteh N, Shojadoost B, Brisbin JT, Emam M, Hodgins DC et al.** Reduction of avian influenza virus shedding by administration of Toll-like receptor ligands to chickens. *Vaccine* 2015;33(38):4843-4849. | No |
| **Beato MS, Realpe-Quintero M, Bonfante F, Mancin M, Ormelli S et al.** Cross-clade protection against H5N1 HPAI strains recently isolated from commercial poultry in Egypt with a single dose of a baculovirus based vaccine. *Vaccine* 2013;31(44):5075-5081. | No |
| **Bertran K, Sa ESM, Pantin-Jackwood MJ, Swayne DE**. Protection against H7N3 high pathogenicity avian influenza in chickens immunized with a recombinant fowlpox and an inactivated avian influenza vaccines. *Vaccine* 2013;31(35):3572-3576. | Yes |
| **Bertran K, Lee D, Balzli C, Pantin-Jackwood MJ, Spackman E et al.** Age is not a determinant factor in susceptibility of broilers to H5N2 clade 2.3.4.4 high pathogenicity avian influenza virus. *Vet Res* 2016;47(116). | Yes |
| **Bertran K, Balzli C, Lee DH, Suarez DL, Kapczynski DR et al.** Protection of White Leghorn chickens by U.S. emergency H5 vaccination against clade 2.3.4.4 H5N2 high pathogenicity avian influenza virus. *Vaccine* 2017. | Yes |
| **Bhatia S, Khandia R, Sood R, Bhat S, Siddiqui A et al.** Reverse genetics based rgH5N2 vaccine provides protection against high dose challenge of H5N1 avian influenza virus in chicken. *Microb Pathog* 2016;97:172-177. | No |
| **Blohm U, Weigend S, Preisinger R, Beer M, Hoffmann D**. Immunological competence of different domestic chicken breeds against avian influenza infection. *Avian diseases*, Conference paper 2016;60(1s):262-268. | No |
| **Bonfante F, Fusaro A, Zanardello C, Patrono LV, De Nardi R et al.** Lethal nephrotropism of an H10N1 avian influenza virus stands out as an atypical pathotype. *Veterinary microbiology* 2014;173(3-4):189-200. | No |
| **Boyd AC, Ruiz-Hernandez R, Peroval MY, Carson C, Balkissoon D et al.** Towards a universal vaccine for avian influenza: protective efficacy of modified Vaccinia virus Ankara and Adenovirus vaccines expressing conserved influenza antigens in chickens challenged with low pathogenic avian influenza virus. *Vaccine* 2013;31(4):670-675. | No |
| **Bublot M, Le Gros FX, Nieddu D, Pritchard N, Mickle TR et al.** Efficacy of two H5N9-inactivated vaccines against challenge with a recent H5N1 highly pathogenic avian influenza isolate from a chicken in Thailand. *Avian diseases* 2007;51(1 Suppl):332-337. | Yes |
| **Bublot M, Pritchard N, Cruz JS, Mickle TR, Selleck P et al.** Efficacy of a fowlpox-vectored avian influenza H5 vaccine against Asian H5N1 highly pathogenic avian influenza virus challenge. *Avian diseases* 2007;51(1 Suppl):498-500. | Yes |
| **Busquets N, Abad FX, Alba A, Dolz R, Allepuz A et al.** Persistence of highly pathogenic avian influenza virus (H7N1) in infected chickens: feather as a suitable sample for diagnosis. *The Journal of general virology* 2010;91(Pt 9):2307-2313. | No |
| **Cagle C, To TL, Nguyen T, Wasilenko J, Adams SC et al.** Pekin and Muscovy ducks respond differently to vaccination with a H5N1 highly pathogenic avian influenza (HPAI) commercial inactivated vaccine. *Vaccine* 2011;29(38):6549-6557. | Yes |
| **Carranza-Flores JM, Padilla-Noriega L, Loza-Rubio E, Garcia-Espinosa G**. Prolonged excretion of a low-pathogenicity H5N2 avian influenza virus strain in the Pekin duck. *Journal of veterinary science* 2013;14(4):487-490. | Yes |
| **Cha RM, Smith D, Shepherd E, Davis CT, Donis R et al.** Suboptimal protection against H5N1 highly pathogenic avian influenza viruses from Vietnam in ducks vaccinated with commercial poultry vaccines. *Vaccine* 2013;31(43):4953-4960. | Yes |
| **Chaves AJ, Busquets N, Campos N, Ramis A, Dolz R et al.** Pathogenesis of highly pathogenic avian influenza A virus (H7N1) infection in chickens inoculated with three different doses. *Avian pathology : journal of the WVPA* 2011;40(2):163-172. | No |
| **Chua TH, Leung CY, Fang HE, Chow CK, Ma SK et al.** Evaluation of a Subunit H5 Vaccine and an Inactivated H5N2 Avian Influenza Marker Vaccine in Ducks Challenged with Vietnamese H5N1 Highly Pathogenic Avian Influenza Virus. *Influenza research and treatment* 2010;2010:489213. | No |
| **Claes G, Welby S, Van Den Berg T, Van Der Stede Y, Dewulf J et al.** The impact of viral tropism and housing conditions on the transmission of three H5/H7 low pathogenic avian influenza viruses in chickens. *Epidemiology and infection* 2013;141(11):2428-2443. | Yes |
| **Claes G, Lambrecht B, Dewulf J, van den Berg T, Marche S**. Extended transmission of two H5/H7 low pathogenic avian influenza viruses in chickens. *Epidemiology and infection* 2015;143(4):781-790. | Yes |
| **Connie Leung YH, Luk G, Sia SF, Wu YO, Ho CK et al.** Experimental challenge of chicken vaccinated with commercially available H5 vaccines reveals loss of protection to some highly pathogenic avian influenza H5N1 strains circulating in Hong Kong/China. *Vaccine* 2013;31(35):3536-3542. | Yes |
| **Dabaghian M, Latify AM, Tebianian M, Nili H, Ranjbar AR et al.** Vaccination with recombinant 4 x M2e.HSP70c fusion protein as a universal vaccine candidate enhances both humoral and cell-mediated immune responses and decreases viral shedding against experimental challenge of H9N2 influenza in chickens. *Veterinary microbiology* 2014;174(1-2):116-126. | No |
| **Das BR, Kumar M, Murugkar HV, Nagarajan S, Kumar DS et al.** Experimental inoculation of a crow derived influenza A (H5N1) virus in chickens and its pathological and genetic characterization. *Indian J Anim Sci* 2016;86(3):238-242. | No |
| **Dash SK, Kumar M, Kataria JM, Nagarajan S, Tosh C et al.** Partial heterologous protection by low pathogenic H9N2 virus against natural H9N2-PB1 gene reassortant highly pathogenic H5N1 virus in chickens. *Microb Pathog* 2016;95:157-165. | No |
| **DeJesus E, Costa-Hurtado M, Smith D, Lee DH, Spackman E et al.** Changes in adaptation of H5N2 highly pathogenic avian influenza H5 clade 2.3.4.4 viruses in chickens and mallards. *Virology* 2016;499:52-64. | Yes |
| **Ducatez MF, Becker J, Freudenstein A, Delverdier M, Delpont M et al.** Low pathogenic avian influenza (H9N2) in chicken: evaluation of an ancestral H9-MVA vaccine. *Veterinary microbiology* 2016;189:59-67. | No |
| **Eggert D, Swayne DE**. Single vaccination provides limited protection to ducks and geese against H5N1 high pathogenicity avian influenza virus. *Avian diseases* 2010;54(4):1224-1229. | Yes |
| **Elaish M, Kang KI, Xia M, Ali A, Shany SA et al.** Immunogenicity and protective efficacy of the norovirus P particle-M2e chimeric vaccine in chickens. *Vaccine* 2015;33(38):4901-4909. | Yes |
| **Guan J, Fu Q, Sharif S**. Replication of an H9N2 Avian Influenza Virus and Cytokine Gene Expression in Chickens Exposed by Aerosol or Intranasal Routes. *Avian diseases* 2015;59(2):263-268. | Yes |
| **Guionie O, Guillou-Cloarec C, Courtois D, Bougeard BS, Amelot M et al.** Experimental infection of Muscovy ducks with highly pathogenic avian influenza virus (H5N1) belonging to clade 2.2. *Avian diseases* 2010;54(1 Suppl):538-547. | Yes |
| **Hassan KE, Ali A, Shany SAS, El-Kady MF**. Experimental co-infection of infectious bronchitis and low pathogenic avian influenza H9N2 viruses in commercial broiler chickens. *Research in veterinary science* 2017;115:356-362. | Yes |
| **Humberd J, Boyd K, Webster RG**. Emergence of influenza A virus variants after prolonged shedding from pheasants. *Journal of virology* 2007;81(8):4044-4051. | Yes |
| **Iqbal M, Yaqub T, Mukhtar N, Shabbir MZ, McCauley JW**. Infectivity and transmissibility of H9N2 avian influenza virus in chickens and wild terrestrial birds. *Vet Res* 2013;44:100. | Yes |
| **Jackwood MW, Suarez DL, Hilt D, Pantin-Jackwood MJ, Spackman E et al.** Biologic characterization of chicken-derived H6N2 low pathogenic avian influenza viruses in chickens and ducks. *Avian diseases* 2010;54(1):120-125. | Yes |
| **Jadhao SJ, Lee CW, Sylte M, Suarez DL**. Comparative efficacy of North American and antigenically matched reverse genetics derived H5N9 DIVA marker vaccines against highly pathogenic Asian H5N1 avian influenza viruses in chickens. *Vaccine* 2009;27(44):6247-6260. | Yes |
| **Jeong OM, Kim MC, Kim MJ, Kang HM, Kim HR et al.** Experimental infection of chickens, ducks and quails with the highly pathogenic H5N1 avian influenza virus. *Journal of veterinary science* 2009;10(1):53-60. | No |
| **Jiang Y, Zhang H, Wang G, Zhang P, Tian G et al.** Protective efficacy of H7 subtype avian influenza DNA vaccine. *Avian diseases* 2010;54(1 Suppl):290-293. | Yes |
| **Jiao P, Song H, Liu X, Song Y, Cui J et al.** Pathogenicity, Transmission and Antigenic Variation of H5N1 Highly Pathogenic Avian Influenza Viruses. *Front Microbiol* 2016:635. | Yes |
| **Kalhoro NH, Veits J, Rautenschlein S, Zimmer G**. A recombinant vesicular stomatitis virus replicon vaccine protects chickens from highly pathogenic avian influenza virus (H7N1). *Vaccine* 2009;27(8):1174-1183. | No |
| **Kalthoff D, Bogs J, Grund C, Tauscher K, Teifke JP et al.** Avian influenza H7N9/13 and H7N7/13: a comparative virulence study in chickens, pigeons, and ferrets. *Journal of virology* 2014;88(16):9153-9165. | No |
| **Kang HM, Lee EK, Song BM, Jeong J, Choi JG et al.** Novel reassortant influenza A(H5N8) viruses among inoculated domestic and wild ducks, South Korea, 2014. *Emerging infectious diseases* 2015;21(2):298-304. | No |
| **Kapczynski DR, Pantin-Jackwood M, Guzman SG, Ricardez Y, Spackman E et al.** Characterization of the 2012 highly pathogenic avian influenza H7N3 virus isolated from poultry in an outbreak in Mexico: pathobiology and vaccine protection. *Journal of virology* 2013;87(16):9086-9096. | Yes |
| **Kapczynski DR, Esaki M, Dorsey KM, Jiang H, Jackwood M et al.** Vaccine protection of chickens against antigenically diverse H5 highly pathogenic avian influenza isolates with a live HVT vector vaccine expressing the influenza hemagglutinin gene derived from a clade 2.2 avian influenza virus. *Vaccine* 2015;33(9):1197-1205. | Yes |
| **Kapczynski DR, Dorsey K, Chrzastek K, Moraes M, Jackwood M et al.** Vaccine Protection of Turkeys Against H5N1 Highly Pathogenic Avian Influenza Virus with a Recombinant Turkey Herpesvirus Expressing the Hemagglutinin Gene of Avian Influenza. *Avian diseases* 2016;60(2):413-417. | Yes |
| **Kapczynski DR, Tumpey TM, Hidajat R, Zsak A, Chrzastek K et al.** Vaccination with virus-like particles containing H5 antigens from three H5N1 clades protects chickens from H5N1 and H5N8 influenza viruses. *Vaccine* 2016;34(13):1575-1581. | Yes |
| **Kilany WH, Ali A, Bazid AH, El-Deeb AH, El-Abideen MA et al.** A Dose-Response Study of Inactivated Low Pathogenic Avian Influenza H9N2 Virus in Specific-Pathogen-Free and Commercial Broiler Chickens. *Avian diseases* 2016;60(1 Suppl):256-261. | Yes |
| **Kilany WH, Bazid AH, Ali A, El-Deeb AH, El-Abideen MA et al.** Comparative Effectiveness of Two Oil Adjuvant-Inactivated Avian Influenza H9N2 Vaccines. *Avian diseases* 2016;60(1 Suppl):226-231. | Yes |
| **Kilany WH, Safwat M, Mohammed SM, Salim A, Fasina FO et al.** Protective Efficacy of Recombinant Turkey Herpes Virus (rHVT-H5) and Inactivated H5N1 Vaccines in Commercial Mulard Ducks against the Highly Pathogenic Avian Influenza (HPAI) H5N1 Clade 2.2.1 Virus. *PloS one* 2016;11(6):Article No. | No |
| **Kim JK, Seiler P, Forrest HL, Khalenkov AM, Franks J et al.** Pathogenicity and vaccine efficacy of different clades of Asian H5N1 avian influenza A viruses in domestic ducks. *Journal of virology* 2008;82(22):11374-11382. | Yes |
| **Kim SM, Kim YI, Park SJ, Kim EH, Kwon HI et al.** Vaccine Efficacy of Inactivated, Chimeric Hemagglutinin H9/H5N2 Avian Influenza Virus and Its Suitability for the Marker Vaccine Strategy. *Journal of virology* 2017;91(6). | Yes |
| **Kim YI, Pascua PN, Kwon HI, Lim GJ, Kim EH et al.** Pathobiological features of a novel, highly pathogenic avian influenza A(H5N8) virus. *Emerging microbes & infections* 2014;3(10):e75. | Yes |
| **Ku KB, Park EH, Yum J, Kim HM, Kang YM et al.** Transmissibility of novel H7N9 and H9N2 avian influenza viruses between chickens and ferrets. *Virology* 2014;450-451:316-323. | Yes |
| **Lardinois A, Steensels M, Lambrecht B, Desloges N, Rahaus M et al.** Potency of a recombinant NDV-H5 vaccine against various HPAI H5N1 virus challenges in SPF chickens. *Avian diseases* 2012;56(4 Suppl):928-936. | No |
| **Li Y, Reddy K, Reid SM, Cox WJ, Brown IH et al.** Recombinant herpesvirus of turkeys as a vector-based vaccine against highly pathogenic H7N1 avian influenza and Marek's disease. *Vaccine* 2011;29(46):8257-8266. | Yes |
| **Londt BZ, Nunez A, Banks J, Nili H, Johnson LK et al.** Pathogenesis of highly pathogenic avian influenza A/turkey/Turkey/1/2005 H5N1 in Pekin ducks (Anas platyrhynchos) infected experimentally. *Avian pathology : journal of the WVPA* 2008;37(6):619-627. | No |
| **Londt BZ, Nunez A, Banks J, Alexander DJ, Russell C et al.** The effect of age on the pathogenesis of a highly pathogenic avian influenza (HPAI) H5N1 virus in Pekin ducks (Anas platyrhynchos) infected experimentally. *Influenza and other respiratory viruses* 2010;4(1):17-25. | No |
| **Lone NA, Spackman E, Kapczynski D**. Immunologic evaluation of 10 different adjuvants for use in vaccines for chickens against highly pathogenic avian influenza virus. *Vaccine* 2017;35(26):3401-3408. | Yes |
| **Maas R, Tacken M, van Zoelen D, Oei H**. Dose response effects of avian influenza (H7N7) vaccination of chickens: serology, clinical protection and reduction of virus excretion. *Vaccine* 2009;27(27):3592-3597. | Yes |
| **Maas R, Rosema S, van Zoelen D, Venema S**. Maternal immunity against avian influenza H5N1 in chickens: limited protection and interference with vaccine efficacy. *Avian pathology: journal of the WVPA* 2011;40(1):87-92. | Yes |
| **Mallick AI, Kulkarni RR, St Paul M, Parvizi P, Nagy E et al.** Vaccination with CpG-adjuvanted avian influenza virosomes promotes antiviral immune responses and reduces virus shedding in chickens. *Viral Immunol* 2012;25(3):226-231. | No |
| **Marche S, Van Borm S, Lambrecht B, Houdart P, van den Berg T**. Chasing notifiable avian influenza in domestic poultry: a case report of low-pathogenic avian influenza h5 viruses in two Belgian holdings. *Transbound Emerg Dis* 2014;61(6):526-536. | No |
| **Mesonero A, Suarez DL, van Santen E, Tang DC, Toro H**. Avian influenza in ovo vaccination with replication defective recombinant adenovirus in chickens: vaccine potency, antibody persistence, and maternal antibody transfer. *Avian diseases* 2011;55(2):285-292. | No |
| **Mondal S, Xing Z, Cardona C**. A comparison of virulence of influenza A virus isolates from mallards in experimentally inoculated turkeys. *Avian diseases* 2013;57(4):790-796. | No |
| **Morales AC, Jr., Hilt DA, Williams SM, Pantin-Jackwood MJ, Suarez DL et al.** Biologic characterization of H4, H6, and H9 type low pathogenicity avian influenza viruses from wild birds in chickens and turkeys. *Avian diseases* 2009;53(4):552-562. | No |
| **Naguib MM, Grund C, Arafa AS, Abdelwhab EM, Beer M et al.** Heterologous post-infection immunity against Egyptian avian influenza virus (AIV) H9N2 modulates the course of subsequent infection by highly pathogenic AIV H5N1, but vaccination immunity does not. *The Journal of general virology* 2017;98(6):1169-1173. | No |
| **Nagy A, Lee J, Mena I, Henningson J, Li Y et al.** Recombinant Newcastle disease virus expressing H9 HA protects chickens against heterologous avian influenza H9N2 virus challenge. *Vaccine* 2016;34(23):2537-2545. | No |
| **Nfon C, Berhane Y, Pasick J, Kobinger G, Kobasa D et al.** Prior infection of chickens with H1N1 avian influenza virus elicits heterologous protection against highly pathogenic H5N2. *Vaccine* 2012;30(50):7187-7192. | No |
| **Niqueux E, Guionie O, Amelot M, Jestin V**. Prime-boost vaccination with recombinant H5-fowlpox and Newcastle disease virus vectors affords lasting protection in SPF Muscovy ducks against highly pathogenic H5N1 influenza virus. *Vaccine* 2013;31(38):4121-4128. | Yes |
| **Nuradji H, Bingham J, Lowther S, Wibawa H, Colling A et al.** A comparative evaluation of feathers, oropharyngeal swabs, and cloacal swabs for the detection of H5N1 highly pathogenic avian influenza virus infection in experimentally infected chickens and ducks. *Journal of veterinary diagnostic investigation : official publication of the American Association of Veterinary Laboratory Diagnosticians, Inc* 2015;27(6):704-715. | No |
| **Ogunremi O, Pasick J, Kobinger GP, Hannaman D, Berhane Y et al.** A single electroporation delivery of a DNA vaccine containing the hemagglutinin gene of Asian H5N1 avian influenza virus generated a protective antibody response in chickens against a North American virus strain. *Clinical and vaccine immunology : CVI* 2013;20(4):491-500. | No |
| **Pan Z, Zhang X, Geng S, Cheng N, Sun L et al.** Priming with a DNA vaccine delivered by attenuated Salmonella typhimurium and boosting with a killed vaccine confers protection of chickens against infection with the H9 subtype of avian influenza virus. *Vaccine* 2009;27(7):1018-1023. | Yes |
| **Pan Z, Zhang X, Geng S, Fang Q, You M et al.** Prime-boost immunization using a DNA vaccine delivered by attenuated Salmonella enterica serovar typhimurium and a killed vaccine completely protects chickens from H5N1 highly pathogenic avian influenza virus. *Clinical and vaccine immunology : CVI* 2010;17(4):518-523. | Yes |
| **Pantin-Jackwood MJ, Smith DM, Wasilenko JL, Spackman E**. Low pathogenicity avian influenza viruses infect chicken layers by different routes of inoculation. *Avian diseases* 2012;56(2): 276-281. | Yes |
| **Pantin-Jackwood M, Swayne DE, Smith D, Shepherd E**. Effect of species, breed and route of virus inoculation on the pathogenicity of H5N1 highly pathogenic influenza (HPAI) viruses in domestic ducks. *Vet Res* 2013;44:62. | Yes |
| **Pantin-Jackwood MJ, Miller PJ, Spackman E, Swayne DE, Susta L et al.** Role of poultry in the spread of novel H7N9 influenza virus in China. *Journal of virology* 2014;88(10):5381-5390. | Yes |
| **Pantin-Jackwood MJ, Kapczynski DR, DeJesus E, Costa-Hurtado M, Dauphin G et al.** Efficacy of a Recombinant Turkey Herpesvirus H5 Vaccine Against Challenge With H5N1 Clades 1.1.2 and 2.3.2.1 Highly Pathogenic Avian Influenza Viruses in Domestic Ducks (Anas platyrhynchos domesticus). *Avian diseases* 2016;60(1):22-32. | Yes |
| **Pantin-Jackwood MJ, Stephens CB, Bertran K, Swayne DE, Spackman E.** The pathogenesis of H7N8 low and highly pathogenic avian influenza viruses from the United States 2016 outbreak in chickens, turkeys and mallards. *PLoS One*. 2017;12(5):e0177265. | No |
| **Park JK, Lee DH, Cho CH, Yuk SS, To EO et al.** Supplementation of oil-based inactivated H9N2 vaccine with M2e antigen enhances resistance against heterologous H9N2 avian influenza virus infection. *Veterinary microbiology* 2014;169(3-4):211-217. | Yes |
| **Peeters B, de Boer SM, Tjeerdsma G, Moormann R, Koch G**. New DIVA vaccine for the protection of poultry against H5 highly pathogenic avian influenza viruses irrespective of the N-subtype. *Vaccine* 2012;30(49):7078-7083. | No |
| **Peeters B, Tonnis WF, Murugappan S, Rottier P, Koch G et al.** Pulmonary immunization of chickens using non-adjuvanted spray-freeze dried whole inactivated virus vaccine completely protects against highly pathogenic H5N1 avian influenza virus. *Vaccine* 2014;32(48):6445-6450. | No |
| **Pfeiffer J, Suarez DL, Sarmento L, To TL, Nguyen T et al.** Efficacy of commercial vaccines in protecting chickens and ducks against H5N1 highly pathogenic avian influenza viruses from Vietnam. *Avian diseases* 2010;54(1 Suppl):262-271. | Yes |
| **Pillai SP, Pantin-Jackwood M, Suarez DL, Saif YM, Lee CW**. Pathobiological characterization of low-pathogenicity H5 avian influenza viruses of diverse origins in chickens, ducks and turkeys. *Arch Virol* 2010;155(9):1439-1451. | Yes |
| **Prel A, Le Gall-Recule G, Cherbonnel M, Grasland B, Amelot M et al.** Assessment of the protection afforded by triple baculovirus recombinant coexpressing H5, N3, M1 proteins against a homologous H5N3 low-pathogenicity avian influenza virus challenge in Muscovy ducks. *Avian diseases* 2007;51(1 Suppl):484-489. | No |
| **Rauw F, Palya V, Gardin Y, Tatar-Kis T, Dorsey KM et al.** Efficacy of rHVT-AI vector vaccine in broilers with passive immunity against challenge with two antigenically divergent Egyptian clade 2.2.1 HPAI H5N1 strains. *Avian diseases* 2012;56(4 Suppl):913-922. | No |
| **Saczynska V, Romanik A, Florys K, Cecuda-Adamczewska V, Kesik-Brodacka M et al.** A novel hemagglutinin protein produced in bacteria protects chickens against H5N1 highly pathogenic avian influenza viruses by inducing H5 subtype-specific neutralizing antibodies. *PloS one* 2017;12(2):e0172008. | Yes |
| **Sasaki T, Kokumai N, Ohgitani T, Sakamoto R, Takikawa N et al.** Long lasting immunity in chickens induced by a single shot of influenza vaccine prepared from inactivated non-pathogenic H5N1 virus particles against challenge with a highly pathogenic avian influenza virus. *Vaccine* 2009;27(38):5174-5177. | Yes |
| **Schroer D, Veits J, Grund C, Dauber M, Keil G et al.** Vaccination with Newcastle disease virus vectored vaccine protects chickens against highly pathogenic H7 avian influenza virus. *Avian diseases* 2009;53(2):190-197. | Yes |
| **Seo SH, Webster RG**. Cross-reactive, cell-mediated immunity and protection of chickens from lethal H5N1 influenza virus infection in Hong Kong poultry markets. *Journal of virology* 2001;75(6):2516-2525. | No |
| **Soejoedono RD, Murtini S, Palya V, Felfoldi B, Mato T et al.** Efficacy of a recombinant HVT-H5 vaccine against challenge with two genetically divergent Indonesian HPAI H5N1 strains. *Avian diseases* 2012;56(4 Suppl):923-927. | No |
| **Song H, Nieto GR, Perez DR**. A new generation of modified live-attenuated avian influenza viruses using a two-strategy combination as potential vaccine candidates. *Journal of virology* 2007;81(17):9238-9248. | Yes |
| **Song JM, Lee YJ, Jeong OM, Kang HM, Kim HR et al.** Generation and evaluation of reassortant influenza vaccines made by reverse genetics for H9N2 avian influenza in Korea. *Veterinary microbiology* 2008;130(3-4):268-276. | Yes |
| **Spackman E, Gelb J, Jr., Preskenis LA, Ladman BS, Pope CR et al.** The pathogenesis of low pathogenicity H7 avian influenza viruses in chickens, ducks and turkeys. *Virology journal* 2010;7:331. | Yes |
| **Spackman E, Pantin-Jackwood MJ, Kapczynski DR, Swayne DE, Suarez DL**. H5N2 Highly Pathogenic Avian Influenza Viruses from the US 2014-2015 outbreak have an unusually long pre-clinical period in turkeys. *BMC veterinary research* 2016;12(1):260. | Yes |
| **St Paul M, Mallick AI, Read LR, Villanueva AI, Parvizi P et al.** Prophylactic treatment with Toll-like receptor ligands enhances host immunity to avian influenza virus in chickens. *Vaccine* 2012;30(30):4524-4531. | No |
| **Steensels M, Van Borm S, Lambrecht B, De Vriese J, Le Gros FX et al.** Efficacy of an inactivated and a fowlpox-vectored vaccine in Muscovy ducks against an Asian H5N1 highly pathogenic avian influenza viral challenge. *Avian diseases* 2007;51(1 Suppl):325-331. | No |
| **Steensels M, Bublot M, Van Borm S, De Vriese J, Lambrecht B et al.** Prime-boost vaccination with a fowlpox vector and an inactivated avian influenza vaccine is highly immunogenic in Pekin ducks challenged with Asian H5N1 HPAI. *Vaccine* 2009;27(5):646-654. | No |
| **Steensels M, Rauw F, van den Berg T, Marche S, Gardin Y et al.** Protection Afforded by a Recombinant Turkey Herpesvirus-H5 Vaccine Against the 2014 European Highly Pathogenic H5N8 Avian Influenza Strain. *Avian diseases* 2016;60(1, Suppl. S):202-209. | Yes |
| **Sylte MJ, Hubby B, Suarez DL**. Influenza neuraminidase antibodies provide partial protection for chickens against high pathogenic avian influenza infection. *Vaccine* 2007;25(19):3763-3772. | Yes |
| **Tavakkoli H, Asasi K, Mohammadi A**. Effectiveness of two H9N2 low pathogenic avian influenza conventional inactivated oil emulsion vaccines on H9N2 viral replication and shedding in broiler chickens. *Iran J Vet Res* 2011;12(3):214-221. | No |
| **Thomas C, Manin TB, Andriyasov AV, Swayne DE**. Limited susceptibility and lack of systemic infection by an H3N2 swine influenza virus in intranasally inoculated chickens. *Avian diseases* 2008;52(3):498-501. | Yes |
| **Tian G, Zeng X, Li Y, Shi J, Chen H**. Protective efficacy of the H5 inactivated vaccine against different highly pathogenic H5N1 avian influenza viruses isolated in China and Vietnam. *Avian diseases* 2010;54(1 Suppl):287-289. | Yes |
| **Tumpey TM, Kapczynski DR, Swayne DE**. Comparative susceptibility of chickens and turkeys to avian influenza A H7N2 virus infection and protective efficacy of a commercial avian influenza H7N2 virus vaccine. *Avian diseases* 2004;48(1):167-176. | Yes |
| **Uchida Y, Kanehira K, Takemae N, Hikono H, Saito T**. Susceptibility of chickens, quail, and pigeons to an H7N9 human influenza virus and subsequent egg-passaged strains. *Arch Virol* 2017;162(1):103-116. | Yes |
| **Umar S, Abdul R, Muhammad Y, Qamar un n, Asif A et al.** Effects of Nigella sativa on immune responses and pathogenesis of avian influenza (H9N2) virus in turkeys. *J Appl Poult Res* 2016;25(1):95-103. | Yes |
| **Umar S, Abdul R, Sajjad A, Muhammad U, Muhammad A et al.** Variation in viral shedding patterns between domestic and wild terrestrial birds infected experimentally with reassortant avian influenza virus (H9N2). *Avian Biol Res* 2016;9(3):200-206. | No |
| **Umar S, Munir MT, Kaboudi K, Rehman A, Asif S et al.** Effect of route of inoculation on replication of avian influenza virus (H9N2) and interferon gene expression in guinea fowl (Numida meleagridis). *Br Poult Sci* 2016;57(4):451-461. | Yes |
| **Umar S, Shah MA, Munir MT, Yaqoob M, Fiaz M et al.** Synergistic effects of thymoquinone and curcumin on immune response and anti-viral activity against avian influenza virus (H9N2) in turkeys. *Poult Sci* 2016;95(7):1513-1520. | Yes |
| **Vergara-Alert J, Moreno A, Zabala JG, Bertran K, Costa TP et al.** Exposure to a low pathogenic A/H7N2 virus in chickens protects against highly pathogenic A/H7N1 virus but not against subsequent infection with A/H5N1. *PloS one* 2013;8(3):e58692. | No |
| **Vergara-Alert J, Busquets N, Ballester M, Chaves AJ, Rivas R et al.** The NS segment of H5N1 avian influenza viruses (AIV) enhances the virulence of an H7N1 AIV in chickens. *Vet Res* 2014;45:7. | No |
| **Wang L, Qin Z, Pantin-Jackwood M, Faulkner O, Suarez DL et al.** Development of DIVA (differentiation of infected from vaccinated animals) vaccines utilizing heterologous NA and NS1 protein strategies for the control of triple reassortant H3N2 influenza in turkeys. *Vaccine* 2011;29(45):7966-7974. | Yes |
| **Yee KS, Carpenter TE, Farver TB, Cardona CJ**. An evaluation of transmission routes for low pathogenicity avian influenza virus among chickens sold in live bird markets. *Virology* 2009;394(1):19-27. | No |
| **Youn HN, Lee YN, Lee DH, Park JK, Yuk SS et al.** Effect of intranasal administration of Lactobacillus fermentum CJL-112 on horizontal transmission of influenza virus in chickens. *Poult Sci* 2012;91(10):2517-2522. | Yes |
| **Yuk S, Erdene-Ochir TO, Kwon J, Noh J, Hong W et al.** Efficacy of clade 2.3.2 H5 commercial vaccines in protecting chickens from clade 2.3.4.4 H5N8 highly pathogenic avian influenza infection. *Vaccine* 2017;35(9):1316-1322. | Yes |
| **Zeng X, Chen P, Liu L, Deng G, Li Y et al.** Protective Efficacy of an H5N1 Inactivated Vaccine Against Challenge with Lethal H5N1, H5N2, H5N6, and H5N8 Influenza Viruses in Chickens. *Avian diseases* 2016;60(1, Suppl. S):253-255. | Yes |
| **Zeng X, Deng G, Liu L, Li Y, Shi J et al.** Protective Efficacy of the Inactivated H5N1 Influenza Vaccine Re-6 Against Different Clades of H5N1 Viruses Isolated in China and the Democratic People's Republic of Korea. *Avian diseases* 2016;60(1, Suppl. S):238-240. | Yes |
| **Zhang Z, Zhang J, Zhang J, Li Q, Miao P et al.** Coimmunization with recombinant epitope-expressing baculovirus enhances protective effects of inactivated H5N1 vaccine against heterologous virus. *Veterinary microbiology* 2017;203:143-148. | Yes |
| **Grey data:** |  |
|  |  |
| **Riks Maas** Wageningen Bioveterinary Research, Lelystad, The Netherlands. Data of HPAI H5N1 | Yes |
| **Jose L Gonzales** Wageningen Bioveterinary Research, Lelystad, The Netherlands. Data of LPAI H7N7. | Yes |
