# Supplementary figures and images for: Virus Shedding of Avian Influenza in Poultry: A Systematic Review and Meta-Analysis
Source: Viruses. 2019 Sep 2;11(9):812. doi: 10.3390/v11090812 (PMC6784017; doi:10.3390/v11090812)

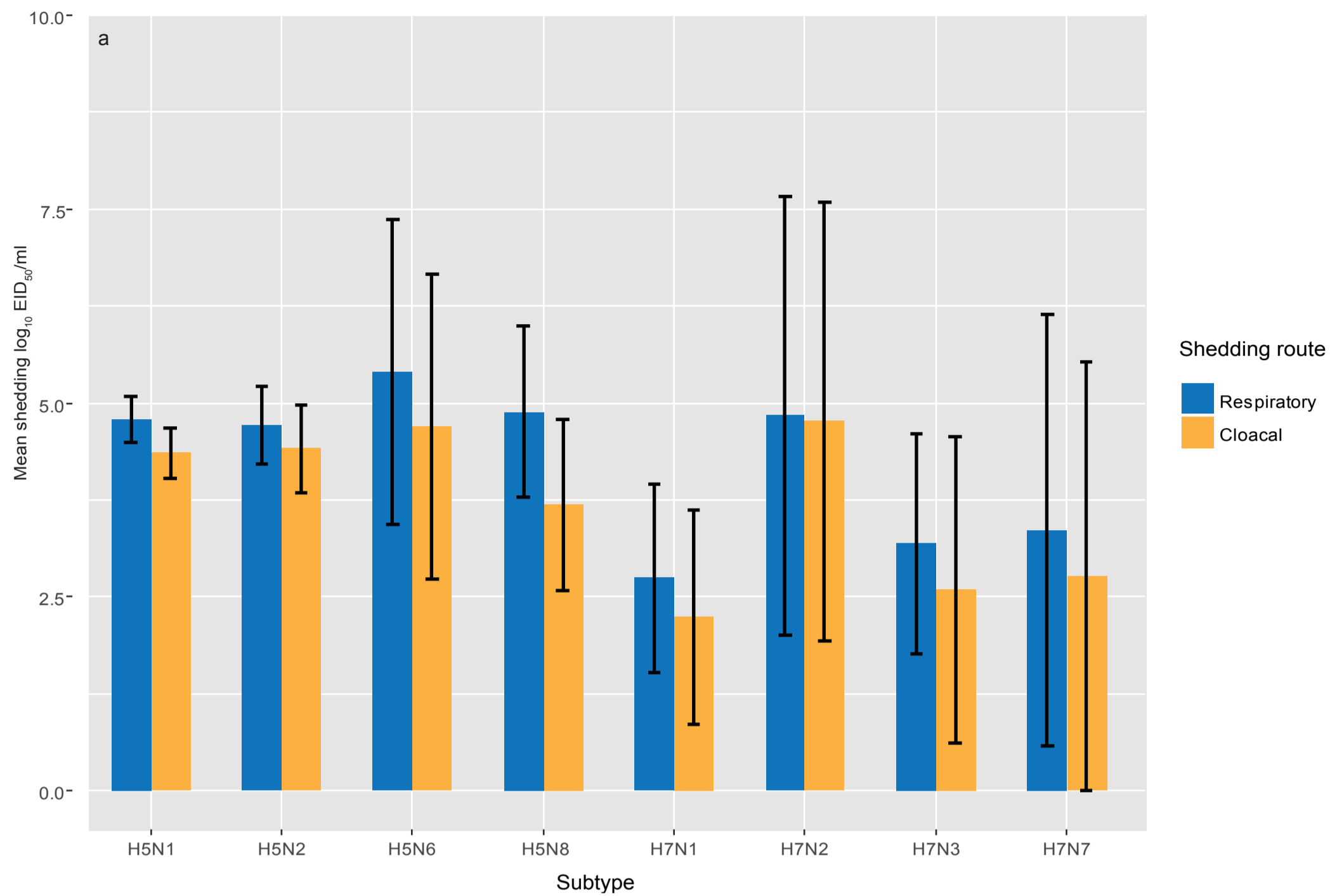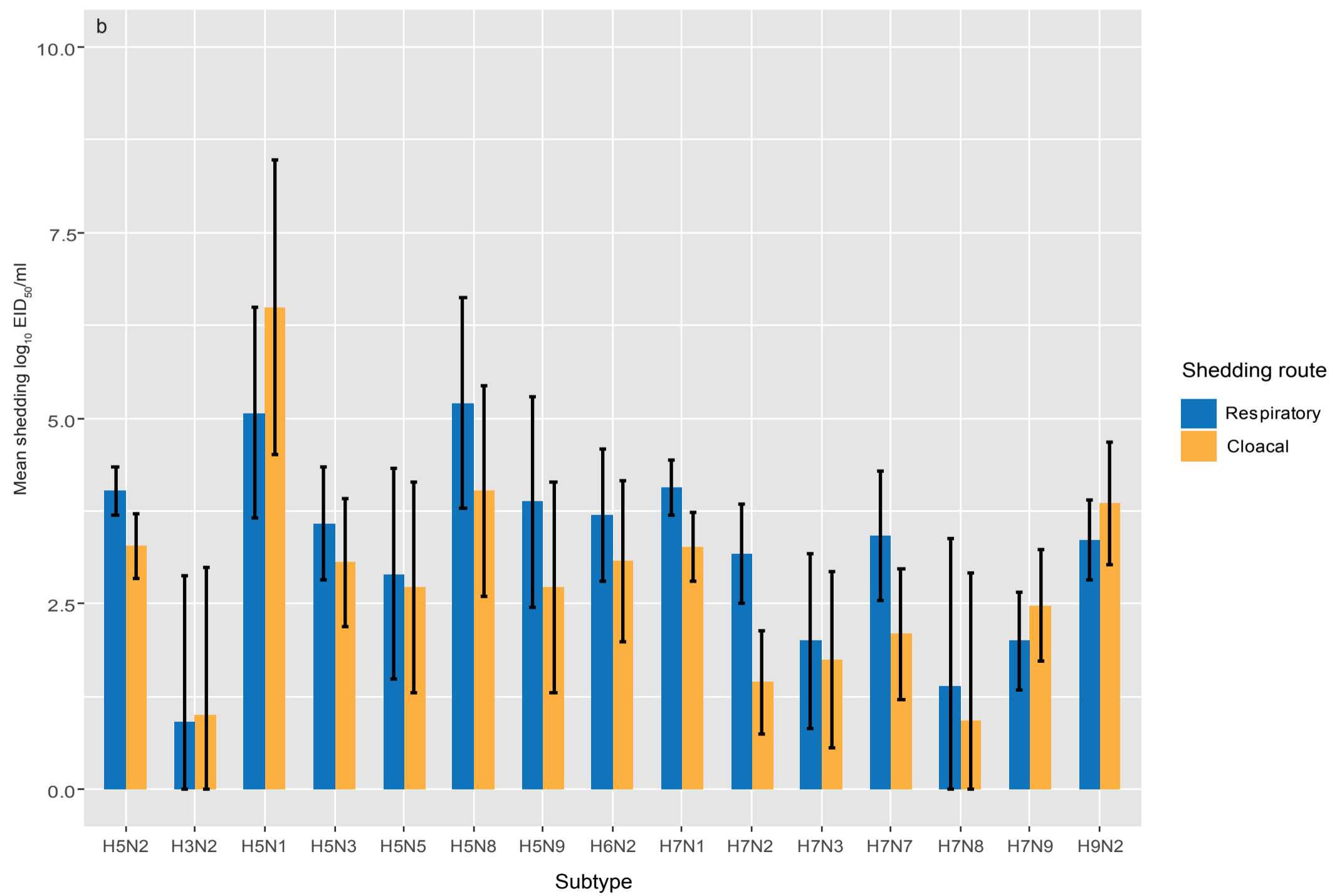

Supplement: Supplementary file 1 [file viruses-11-00812-s001.zip › AI_Shedding_Suppl_Figure1.pdf]
